# Improved osmotic energy conversion in heterogeneous membrane boosted by three-dimensional hydrogel interface

**DOI:** 10.1038/s41467-020-14674-6

**Published:** 2020-02-13

**Authors:** Zhen Zhang, Li He, Congcong Zhu, Yongchao Qian, Liping Wen, Lei Jiang

**Affiliations:** 1 Key Laboratory of Bio-inspired Materials and Interfacial Science, Technical Institute of Physics and Chemistry, Chinese Academy of Sciences, Beijing, 100190 China; 2 Beijing National Laboratory for Molecular Sciences (BNLMS), Key Laboratory of Green Printing, Institute of Chemistry, Chinese Academy of Sciences, Beijing, 100190 China; 3 University of Chinese Academy of Sciences, Beijing, 100049 China; 4 Present Address: Center for Advancing Electronics Dresden (Cfaed) and Faculty of Chemistry and Food Chemistry, Technische Universität Dresden, 01062 Dresden, Germany

**Keywords:** Devices for energy harvesting, Materials for energy and catalysis, Nanofluidics

## Abstract

The emerging heterogeneous membranes show unprecedented superiority in harvesting the osmotic energy between ionic solutions of different salinity. However, the power densities are limited by the low interfacial transport efficiency caused by a mismatch of pore alignment and insufficient coupling between channels of different dimensions. Here we demonstrate the use of three-dimensional (3D) gel interface to achieve high-performance osmotic energy conversion through hybridizing polyelectrolyte hydrogel and aramid nanofiber membrane. The ionic diode effect of the heterogeneous membrane facilitates one-way ion diffusion, and the gel layer provides a charged 3D transport network, greatly enhancing the interfacial transport efficiency. When used for harvesting the osmotic energy from the mixing of sea and river water, the heterogeneous membrane outperforms the state-of-the-art membranes, to the best of our knowledge, with power densities of 5.06 W m^−2^. The diversity of the polyelectrolyte and gel makes our strategy a potentially universal approach for osmotic energy conversion.

## Abstract (web summary)

Heterogeneous membranes show great promise in harvesting the osmotic energy, but the performance is limited by the low interfacial transport efficiency. Here, the authors report use of a three-dimensional polyelectrolyte gel interface to achieve high-performance osmotic energy conversion.

## Introduction

The ionic gradient between fresh water and sea water has been identified as a promising source of renewable energy, also known as blue and osmotic energy, or salinity gradient energy in engineering community^1,2^. In the past decades, membrane-based reverse electrodialysis has been developed as a main technology to capture this worldwide energy^3–7^. However, the current reverse electrodialysis technology generally exhibits a low efficiency, which is mostly due to the low performance of the ion-exchange membranes in use, impeding its real-world applications. Through millions of years’ evolution, the living organisms have developed many optimized systems for power generation from such type of ionic gradients. For example, the electric eel is able to accomplish the conversion of ion concentration gradient across the cellular membrane into the release of action potentials of up to 600 V using thousands of densely packed, highly selective, and rectifying ion channels^8^. Through combining inspiration from living systems with recent advances in nanofluidics, the bioinspired nanofluidic channels are exhibiting a huge potential in harvesting this “blue” energy. Previously, explorations with single-pore- and few-pore-based nanofluidic devices such as boron nitride, molybdenum disulfide, and silica clearly indicate that the integration of a new class of materials for nanofluidic channel with tailored ion transport dynamics will largely boost the osmotic conversion^9–13^, which are currently inspiring scientists to construct macroscopic-scale porous nanofluidic membranes with the purpose for further applications^14,15^.

In this respect, the emerging bioinspired nanofluidic heterogeneous membranes that are constructed by the hybridization of two monolayer porous membranes have shown unprecedented superiority^16–20^. The synergistic effect between the two layers with inherent asymmetric structure, charge, and wettability would contribute to novel ionic transport properties such as ionic diode effect^21–23^. This nonlinear nanofluidic behavior allows unidirectional ion transport and thus could prohibit the flow of any current back into membrane during the energy conversion process, largely decreasing the dissipation of Gibbs free energy as Joule heat and ultimately enhancing the power generation^14,24–26^. In the past years, diverse heterogeneous membranes including organic/organic, inorganic/inorganic, and organic/inorganic hybrid system have been constructed^27–31^. However, the reported system generally suffers from a relatively low ion transport efficiency in the interface (i.e., transition region in the junction of the two layers of membranes) caused by a mismatch of pore alignment and inappropriate coupling between channels of different dimensions, leading to economically unviable power densities. The insufficient interfacial transport has been a main obstacle for the development of high-power heterogeneous membranes^32^.

Herein, we demonstrate the first use of a three-dimensional (3D) hydrogel interface to achieve high-performance osmotic energy conversion. An organic heterogeneous membrane composed of one layer of functional polyelectrolyte hydrogel membrane and one layer of supporting porous aramid nanofiber (ANF) membrane is constructed through a sequential blade-casting method. The inherent electrostatic, chemical, and structural asymmetries render the system stable ionic diode effect, greatly facilitating the cation transport from the nanofiber layer to the hydrogel layer. Furthermore, the hydrogel membrane can provide a widely charged 3D network for ion diffusion and thus could greatly enhance the interfacial transport efficiency, which sheds light on the salinity gradient power generation. When mixing natural sea water and river water, the power output can achieve a very high value ~5.06 W m^−2^, outperforming the state-of-the-art membranes to the best of our knowledge. This work, as an example, demonstrates the great promise of polyelectrolyte gel as high-performance interfacial materials in designing heterogeneous membrane-based osmotic power generators.

## Results

### Fabrication of the heterogeneous membrane

**Fig. 1:**
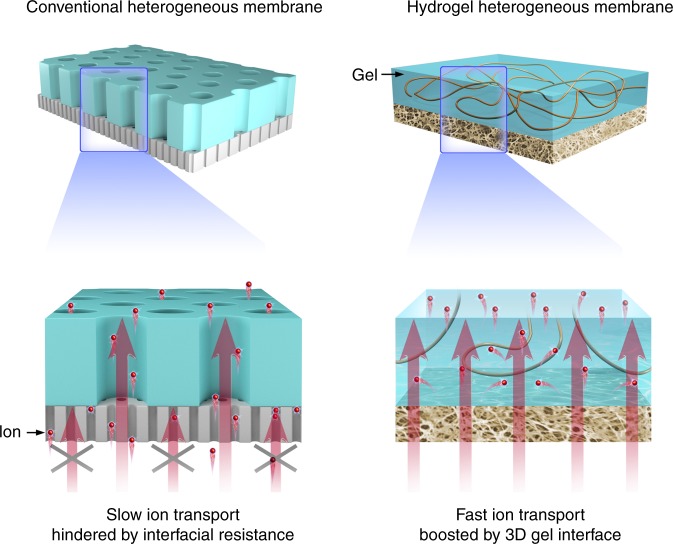
Three-dimensional hydrogel interface with high transport efficiency. The conventional heterogeneous membrane suffers from low interfacial ion transport efficiency caused by mismatch of pore alignment and insufficient coupling between channels of different dimensions. Polyelectrolyte hydrogel-based heterogeneous membrane featuring 3D gel interface with high ion transport efficiency can substantially boost the osmotic energy conversion.

**Fig. 2:**
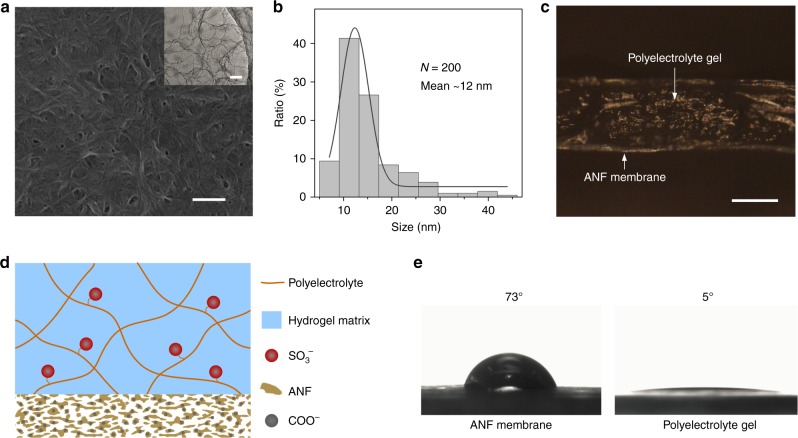
Characterization of the heterogeneous membrane. **a** Surface SEM image of the ANF membrane (scale bar: 300 nm). The inset shows TEM image of the ANFs (scale bar: 250 nm). **b** Pore-size distribution of the ANF membrane obtained through data statistics of the SEM image (the gray line represents the Gauss fit). **c** Photograph of the cross-section view of the ANF/gel heterogeneous membrane (scale bar: 100 μm). **d** 2D cross-section illustration of the hybrid membrane featuring asymmetric structure and charge distribution. **e** Contact angle measurements of the ANF membrane and polyelectrolyte gel.

The heterogeneous membrane is fabricated through hybridizing polyelectrolyte hydrogel membrane and ANF membrane (Fig. 1). The extremely strong Kevlar yarns (Supplementary Fig. 1) are composed of numerous long molecular chains linked by the intermolecular hydrogen bonds. The reaction of Kevlar with dimethyl sulfoxide (DMSO) and potassium hydroxide (KOH) mixtures will weaken the hydrogen bonds and increase the electrostatic repulsion between the polymer chains, chemically transforming the bulk Kevlar macroscale fibers into nanofibers (i.e., ANFs)^33^. The obtained nanofiber dispersion shows a red color that contains a large number of ANFs with length in micrometer scale and diameter in the range of 5–10 nm (Fig. 2a inset and Supplementary Fig. 2). Next, the ANF membranes are prepared through blade-casting followed by a solvent exchange process (see Methods)^34^. The as-prepared ANF membrane is interwoven by numerous nanofibers and the gaps formed by the stacked neighboring nanofibers constitute ion transporting pores (Fig. 2a). Statistical analysis indicates that the mean size of the pore is ~12 nm that is the same order of magnitude as the constituent nanofiber (Fig. 2b). To fabricate the hybrid membrane, hot agarose/polyelectrolyte (poly(sodium 4-styrenesulfonate), PSS) solution is coated onto the ANF membrane by the same blade-casting method and then cooled down at ambient temperature, resulting in a uniform coating of polyelectrolyte gel layer with thickness ranging from several ten to hundred micrometers. As shown in the cross-sectional micro-image (Fig. 2c), the thickness of the gel layer is much larger than that of the ANF membrane (~2 μm) (Supplementary Fig. 3). Benefiting from the introduction of ultrastrong ANF membrane, the heterogeneous membrane exhibits a high mechanical strength with a tensile strength ~35 MPa (Supplementary Fig. 4). The hybrid system has inherent asymmetric structure, charge distribution, and wettability (Fig. 2d). In detail, the ANF membrane is hydrophilic and exhibits nanosized pore dimension. The chemical transforming process will also create functional carboxyl groups on the ANFs^35^, as confirmed by the high-resolution X-ray photoelectron spectroscopy analysis (Supplementary Fig. 5). Because of the existence of these carboxyl groups, the ANF membrane is negatively charged in neutral solution with a Zeta potential about −37 mV (Supplementary Fig. 6). With respect to the gel layer, the polyelectrolytes possess a negative charge due to the rich sulfonate group with a Zeta potential about −25 mV (Supplementary Fig. 7)^36,37^. The gel matrix behaves in a super-hydrophilic nature and could provide much wider 3D ion transport routes (Fig. 2e).

### Charge-governed asymmetric ion transport

**Fig. 3:**
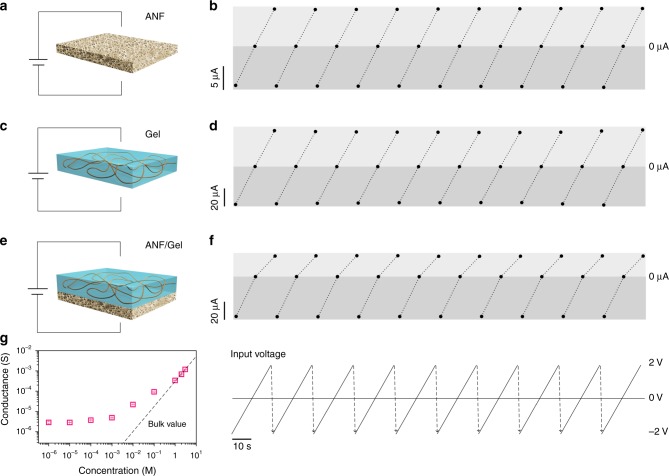
Charge-governed asymmetric ion transport. **a**, **b** *I*–*V* curves of the separate ANF membrane under a symmetric voltage of ±2 V. **c**, **d** *I*–*V* curves of the separate polyelectrolyte gel membrane under a symmetric voltage of ±2 V. **e**, **f** *I*–*V* curves of the heterogeneous membrane under a symmetric voltage of ±2 V. The cathode is placed in the ANF side and the testing electrolyte solutions for the above three conditions are all 0.01 M KCl (pH ~ 7). **g** The transmembrane ionic conductance deviates remarkably from the bulk value (dashed line) when the electrolyte concentration is <0.1 M, indicating a charge-governed ionic transport. Error bars represent SD (*n* = 3).

The ionic transport properties of the heterogeneous membrane are examined by measuring the transmembrane current under symmetric electrolyte condition (0.01 M KCl pH ~ 7) with a custom-made conductivity cell (Supplementary Fig. 8)^38^. Figure 3a, b shows a series of measured current–voltage (*I*–*V*) curves of the separate ANF membrane under a symmetric voltage of ±2 V. The *I*–*V* response is linear with an ionic current ~8 μA at −2 V and remains stable in the testing condition (Supplementary Fig. 9). Similar to the separate ANF membrane, the *I*–*V* response of the separate polyelectrolyte gel is also linear, but with a larger ionic current of about 40 μA (Fig. 3c, d). The enhanced current could be ascribed to its super-hydrophilicity and much wider ion transport route. The separate ANF and gel membranes both behave as Ohmic conductors, as they are negatively charged and have symmetric structure^39^. The situation is different for the hybrid membrane. As shown in Fig. 3e, f, the *I*–*V* response of the heterogeneous system is nonlinear and the system exhibits obvious ionic rectifying behavior. This is because the inherent electrostatic, chemical, and structural asymmetries would contribute to asymmetric flow of ionic species upon switching the external voltage^40^. Notably, the coating of extra ANF layer onto the hydrogel membrane has not largely decreased, but even enhances the ion transporting ability with an ionic current ~44 μA (at −2 V). Under negative bias, the elevated electric potential^41^ inside the asymmetric membrane contributes to enhanced electrostatic interaction between the ions and the surface charge of the nanochannel, resulting in enhanced transmembrane ionic current. The electrolyte concentration also has a clear influence on the transmembrane ionic conductance (Fig. 3g). As the concentration decreases, the measured ionic conductance gradually deviates from the bulk value (dashed line, linear fit of the conductance measured at high concentration region^42^) at 0.1 M, indicating that the transmembrane ionic transport process is fully governed by the surface charge^43^.

### Osmotic energy conversion behavior

**Fig. 4:**
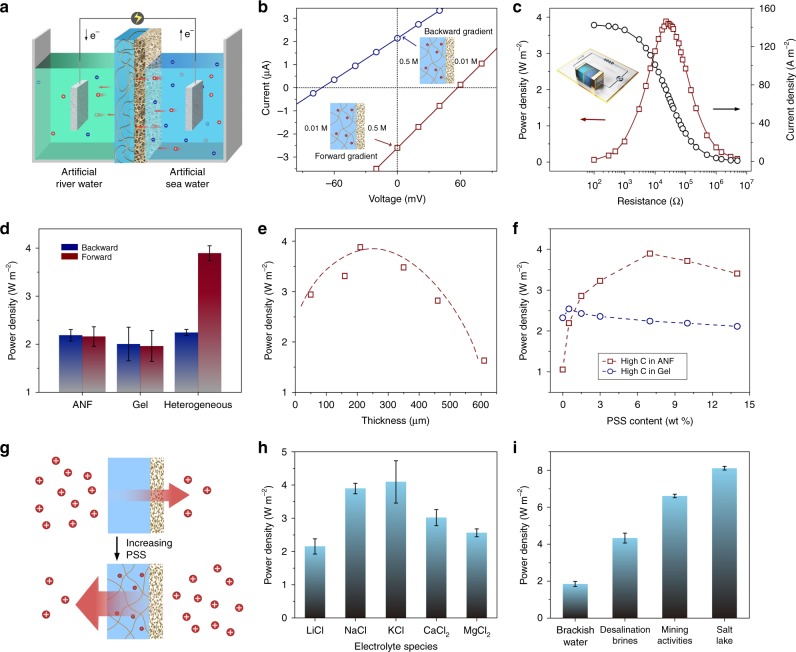
Osmotic energy conversion behavior. **a** Schematic of the osmotic energy conversion process. **b** *I*–*V* curves of the hybrid membrane under forward and backward NaCl concentration gradient. **c** The power generation on the external load under forward concentration gradient. Inset: schematic illustration of the experimental setup for the measurement of osmotic power with electrical resistor. **d** Comparison of the power density of the separate ANF membrane, gel membrane, and the heterogeneous membrane when mixing 0.5 M/0.01 M NaCl. **e**, **f** Influence of the thickness of the polyelectrolyte hydrogel membrane (**e**) and the content of polyelectrolyte (**f**) on the power density. The concentration gradient is 0.5 M/0.01 M NaCl. **g** Schematic of the reversion of dominant diffusion direction upon increasing the dosage of polyelectrolyte. **h** Power generation under a series of electrolyte species. **i** Power generation under a series of artificial water resources including brackish water, desalination brines, brines from mining activities, and water from salt lake. Error bars represent SD (*n* = 3).

To evaluate the osmotic energy conversion performance, artificial sea water (0.5 M NaCl) and river water (0.01 M NaCl) were applied across the heterogeneous membrane (Fig. 4a). As the gel and the ANF membranes are both negatively charged in the testing solution, the concentration gradient will induce a net flow of cations (i.e., Na^+^) across the hybrid membrane, generating the diffusion potential and diffusion current. Two configurations can be adopted to arrange the concentration gradient (Fig. 4b). When the low concentration solution is placed in the gel side, the short-circuit current and open-circuit voltage is ~2.61 μA and 57.2 mV, respectively (the contribution of redox potential on the electrode has been subtracted; Supplementary Figs. 10, 11). Under a reverse concentration gradient with low concentration in the ANF side, the corresponding short-circuit current and open-circuit voltage change to 2.14 μA and 71.6 mV, and the calculated inner resistance increases by ~50%, which largely suppresses the power generation performance. These observation indicates that the preferential direction of ion transport caused by ionic diode effect still exists under a salinity gradient^25^. Hereafter, the concentration gradient where high concentration is located in the ANF side is defined as the forward gradient, whereas the one where high concentration is located in the gel side is defined as the backward gradient. The extracted electric power can also be used to supply an electrical load resistor in an external circuit. The current density decreases upon increasing the external resistance, the output power density achieves a maximum value about 3.9 W m^−2^ at an intermediate resistance about 23 kΩ (Fig. 4c), and the energy conversion efficiency is calculated to be 19.2% (Supplementary Note 1)^10^. Notably, the separate ANF and gel membranes both show relatively low power output of about 2 W m^−2^ and are irrespective of the direction of the concentration gradient (Fig. 4d and Supplementary Fig. 12). Such a directional and high-performance power output could be ascribed to the synergistic effect of the two functional layers. The nonlinear (ionic diode–type) current allows one-way diffusion from the nanofiber side to the hydrogel side, largely decreasing the ion polarization that causes the loss of Gibbs free energy as Joule heating^14^. In addition, the wide 3D charged network of gel membrane will substantially enhance the interfacial transport, contributing to such a considerable power density. The thickness of the gel membrane has substantial influence on the energy conversion behavior. The power output achieves a maximum value at an intermediate thickness of about 210 μm (Fig. 4e). For a relatively thin gel layer, the functional groups are insufficient to achieve an optimal functionality. For a relatively thick gel layer, the transmembrane ion resistance will increase drastically, leading to a decrease in the power output.

We systematically investigate the influence of the PSS content in the gel layer on the output power density (Fig. 4f). For the forward concentration gradient, the power density increases from 1.1 to 3.9 W m^−2^ as the content of PSS increases from 0 to 7%, which can be ascribed to the increased charge density in the gel layer. Continuously increasing the PSS content to 14% leads to a decrease of power densities. In this case, the excess PSS chains will introduce large steric hindrance to the transmembrane ion transport, resulting in a decreased ionic flux and power density. For the backward concentration gradient, the power density undergoes a slight increase from 2.3 to 2.5 W m^−2^ upon increasing the PSS content to 0.5%. Continuously increasing the PSS content results in a decrease of power density to 2.1 W m^−2^. We also note that the dominant diffusion direction will reverse upon changing the dosage of polyelectrolyte (Fig. 4g). When there is no polyelectrolyte in the gel, the gel actually acts as a blockage for ion transport due to the low ion diffusion rate and thus dominates the diffusion direction^44^. The transmembrane transport of ions from the gel layer to the ANF layer is more favorable. After doping with polyelectrolyte, due to the effect of confined electric double layer, the highly charged network will act as an accelerator for ion transport. The ANF layer with smaller pore size will contribute to the main resistance and dominate the diffusion direction.

The energy conversion behaviors of the heterogeneous membrane with various electrolyte are also tested separately. As shown in Fig. 4h, for the monovalent ions, the system behaves maximum power output for the KCl (~4.1 W m^−2^) and minimum power output for the LiCl (~2.15 W m^−2^), because the diffusion coefficient of K^+^ (1.960 × 10^−9^ m^2^ s^−1^) is the largest and the diffusion coefficient of Li^+^ (1.03 × 10^−9^ m^2^ s^−1^) is the smallest. The faster the cation diffuses, the more efficient the charge separation will occur, which is a typical behavior of cation-selective membranes^45,46^. The system also exhibits considerable power output for the divalent ions such as Ca^2+^ and Mg^2+^ ions (i.e., 3.0 and 2.6 W m^−2^, respectively), which is different from the traditional ion exchange membrane-based reverse electrodialysis where the negative effects of divalent ion such as uphill transport, chelation, and electrostatic loading will largely undermine the power output^47^. Notably, although the diffusion coefficient of Ca^2+^ (0.793 × 10^−9^ m^2^ s^−1^) and Mg^2+^ (0.705 × 10^−9^ m^2^ s^−1^) are lower than that of Li^+^ (1.03 × 10^−9^ m^2^ s^−1^), the obtained power densities are much larger that in LiCl electrolyte, which can be ascribed to the stronger electrostatic interaction due to their large intrinsic charge. Furthermore, we find that the performance of the heterogeneous membrane generator would enable it to collect energy using diverse types of feed water resource in nature and industrial activities. For example, replacing the artificial sea water with artificial brackish water (12 g L^−1^), desalination brine (75 g L^−1^), mining waste water (175 g L^−1^), and salt lake water (250 g L^−1^) will contribute to power densities ~1.9, 4.3, 6.6, and 8.1 W m^−2^, respectively (Fig. 4i)^48^. However, the working performance of the membrane will be influenced by the external pH value. A decrease of power density in acidic and alkaline ionic solutions is observed (Supplementary Fig. 13).

**Fig. 5:**
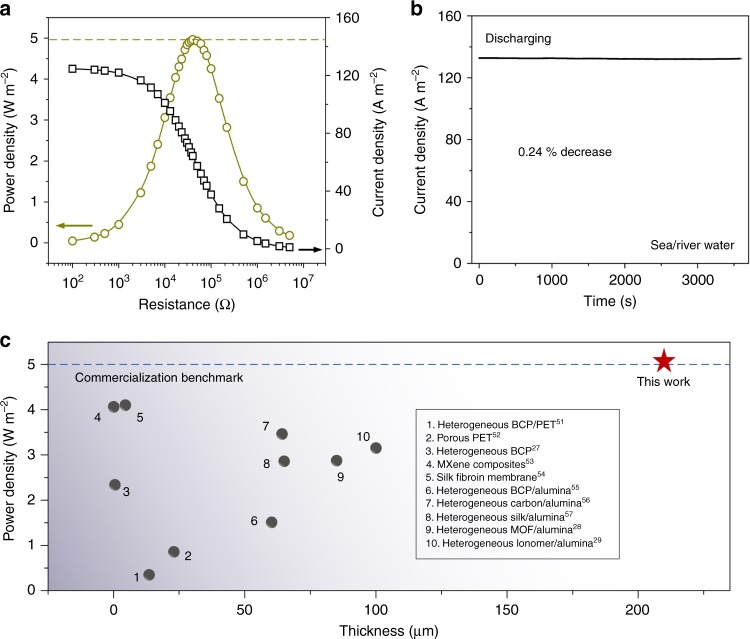
Enhanced osmotic energy conversion with natural water source. **a** When we mix the real sea water with river, the power density could achieve ~4.97 W m^−2^. Data statistics indicate the average power density are 5.06 ± 0.33 W m^−2^. **b** Without electrolyte solution replenishing, the corresponding diffusion current only undergoes 0.24% decrease in an hour, indicating that the heterogeneous membrane can efficiently stabilize local concentration gradient and promote continuous osmotic energy conversion. **c** Comparison of osmotic energy conversion performance with the reported macroscopic-scale porous nanofluidic membranes based on the same testing area.

To examine the application viability of the newly designed heterogeneous membrane in real condition, natural sea water and river water is mixed. As the external resistance increases, the current density decreases accordingly and the maximum power density could achieve ~4.97 W m^−2^ (Fig. 5a and Supplementary Fig. 14). Data statistics indicate the average power density are 5.06 ± 0.33 W m^−2^ (Supplementary Fig. 15). Without electrolyte replenishing, the short-circuit current only exhibits 0.24% attenuation in an hour, indicating that the heterogeneous can efficiently stabilize local concentration gradient and promote continuous osmotic energy harvesting (Fig. 5b). The salinity of sea water and river water is analyzed by ionic conductivity measurement to be 0.4 M and 0.003 M NaCl (see Methods), respectively. The high power output can be ascribed to the co-existence of diverse types of salt contents such as Na^+^, K^+^, Mg^2+^, and Ca^2+^ in the sea water and also the larger salinity gradient difference between the two types of natural water (~130-fold). Compared with pressure-retarded osmosis technology^49,50^, the power density is not higher. However, to the best of our knowledge, this power density is the highest value reported in macroscopic-scale porous nanofluidic membrane-based reverse electrodialysis (Fig. 5c)^27–29,51–57^ and reaches the commercialization benchmark (5 W m^−2^)^58,59^. It should be noted that the testing membrane area is small. It is experimentally challenging to scale up the heterogeneous membrane, which can be ascribed to the combined effect of multiple factors such as enhanced entering resistance (i.e., the sum of reservoir resistance and reservoir/nanopores interfacial resistance), hindered counter-ion diffusion (caused by relatively low selectivity), and increased stochastic defects. Normalizing this high power density into real high power for industrial large-scale membrane applications is a necessity, which needs the joint efforts of both experimental and theoretical scientists in the future^60,61^.

## Discussion

In summary, we observed high-performance osmotic energy conversion in a newly designed heterogeneous membrane prepared by coating charged polyelectrolyte hydrogel onto ANF membrane. Due to the inherent multi-level asymmetries, the hybrid membrane exhibits charge-governed asymmetric ion transport behavior, which can largely decrease the ion polarization phenomenon. Moreover, the polyelectrolyte gel with wide charged 3D network can act as an ion diffusion accelerator that greatly improves the interfacial transport efficiency. Such a membrane design substantially facilitates the transmembrane ionic diffusion, contributing to a high power density of 5.06 W m^−2^ which, to the best of our knowledge, is the highest value reported in nanofluidic membrane-based osmotic energy conversion. Furthermore, good tunability in the functionality of the polyelectrolyte hydrogel membrane allows for systematical understanding of the controllable ion diffusion mechanism and its influence on the overall membrane performance. This work highlights the importance of interface design on the construction of nanofluidic membrane-based osmotic energy conversion system and also provides a basic example that could potentially spark further experimental and theoretical efforts for the application of versatile polyelectrolyte gel materials in this area.

## Methods

### Chemicals

PSS (*M*_w_ ~ 70,000) and high-purity agarose was purchased from Sigma-Aldrich. Kevlar yarn was purchased from Dupont. Analytical grade KCl, NaCl, LiCl, CaCl_2_, MgCl_2_, and KOH was purchased from J&K Beijing Co., Ltd. Sea water was obtained from the Yellow Sea (Qingdao).

### Fabrication of ANF membrane

First, the ANF was prepared by a chemical exfoliation process. Bulk Kevlar yarn (0.5 g) and 0.5 g KOH were added into 50 mL of DMSO. The mixture was magnetically stirred for 1 week at room temperature, resulting in a viscous dark red ANF dispersion. Then a thin layer of ANF dispersion was fabricated on glass substrate by a blade-casting method. After immersing the glass substrate into deionized water, the DMSO can be removed by a solvent-exchange process, forming an ANF liquid membrane that can spontaneously separate from the substrate. The as-prepared ANF liquid membrane was clamped between two pieces of polytetrafluoroethylene (PTFE) board. After drying in 80 °C for 1 day, ultrathin porous membrane interwoven by ANFs can be fabricated. The pore size distribution of the ANF membrane was obtained through data statistics of the corresponding surface scanning electron microscope (SEM) image^62^.

### Fabrication of the composite membrane

PSS solutions with different weight ratio were prepared with ultrapure water. Then, agarose (4.0 wt %) was added into the polyelectrolyte solution and the mixture was boiled on a heating stage under continuous stirring. The hybrid membrane is prepared through direct blade-casting hot agarose/polyelectrolyte solution onto the ANF membrane. The gelation of the solution at ambient temperature will result in a uniform coating of polyelectrolyte hydrogel membrane. The thickness of the hydrogel layer can be precisely tuned by regulating the blade position.

### Electric measurement

The as-prepared ANF and polyelectrolyte hydrogel hybrid membrane was mounted between two chambers of a custom-made electrochemical cell. The ion transport measurements were performed with a Keithley 6487 semiconductor picoammeter (Keithley Instruments, Cleveland, OH). The working electrode was placed in the polyelectrolyte hydrogel membrane side and ground electrode was placed in the ANF membrane side. A pair of homemade Ag/AgCl electrodes was used to collect the current and voltage signals. The subsequent energy conversion tests were performed by connecting the power source with an adjustable resistance box (Zhengyangxing, Shenzhen, China). For a given resistance (*R*_L_), the output power density can be calculated as *P* = *I*^2^ × *R*_L_. The effective testing membrane area was about 3 × 10^4^ µm^2^, which was set to be consistent with the previous reports^56^. In this work, the conductivities of the natural sea water and river water are 36,500 and 442 us cm^−1^ (with Ohaus ST300C Starter conductivity meter), respectively, and the corresponding salt concentration is calibrated with a standard curve obtained by measuring the conductivity of a series of standard NaCl with known concentration. All electrolyte solutions were prepared using Milli-Q water (18.2 MΩ cm^−1^). The pH values of electrolyte solutions were adjusted using HCl and NaOH solutions.

## Supplementary information

Supplementary Information

## Data Availability

The data that supports the findings of this study are available from the corresponding author upon reasonable request.
