# Supplementary material for: Improved osmotic energy conversion in heterogeneous membrane boosted by three-dimensional hydrogel interface
Source: Nat Commun. 2020 Feb 13;11:875. doi: 10.1038/s41467-020-14674-6 (PMC7018769; doi:10.1038/s41467-020-14674-6)
Supplement: Supplementary file 1 — Supplementary Information [file 41467_2020_14674_MOESM1_ESM.pdf]

Supplementary Information

Improved osmotic energy conversion in heterogeneous membrane boosted by three-dimensional hydrogel interface

Zhang *et al.*

Supplementary Note 1. Calculation of energy conversion efficiency

The energy conversion efficiency of the osmotic energy conversion system is defined as the ratio of the output energy (electrical energy) to the input energy (Gibbs free energy of mixing), and can be calculated as[1]:

$$\eta_{\max} = \frac{1}{2}(2t_+ - 1)^2$$

The value of t_+ can be given as:

$$t_+ = \frac{1}{2}\left(\frac{E_{diff}}{\frac{RT}{zF}\mathrm{In}\left(\frac{r_{c_H} c_H}{r_{c_L} c_L}\right)} + 1\right)$$

where E_{diff}, R, T, F, z, γ, and c refer to the diffusion potential, universal gas constant, absolute temperature, Faraday constant, charge number, activity coefficient of ions, ion concentration, respectively.

Supplementary Figures

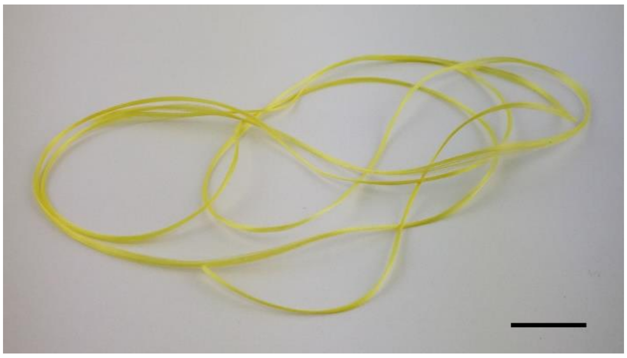

Supplementary Figure 1. Photograph of the Kevlar fibers (scale bar: 1 cm).

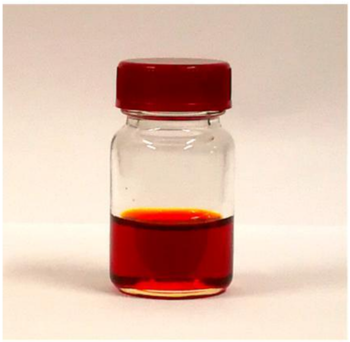

Supplementary Figure 2. Photograph of the ANF dispersion.

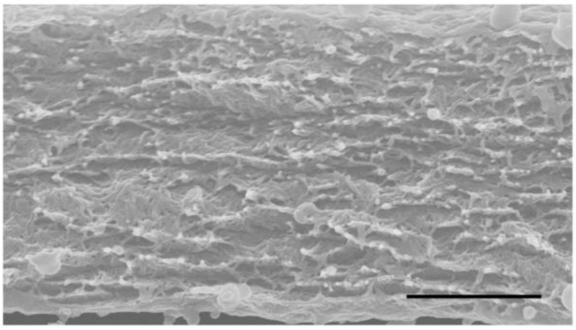

Supplementary Figure 3. Cross-section SEM image of the ANF membrane (scale bar: 1 μm).

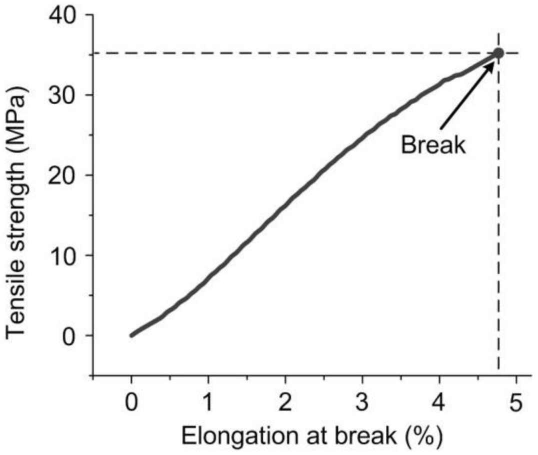

Supplementary Figure 4. The stress–strain curve of the heterogeneous membrane in dry state.

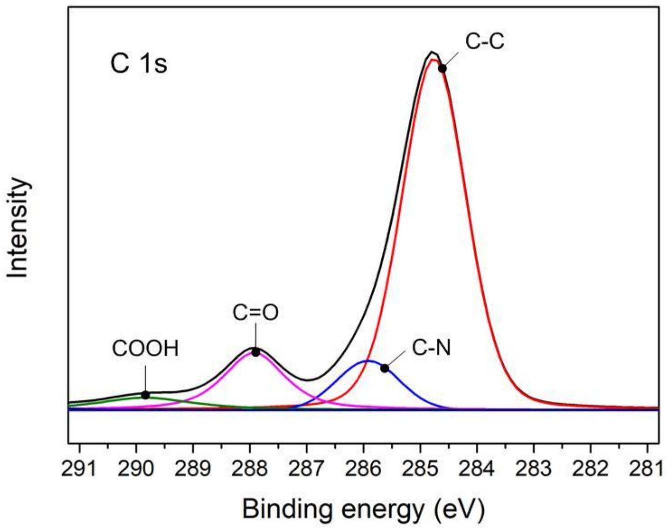

Supplementary Figure 5. C 1s XPS spectra of the ANF membrane, implying the existence of functional carboxyl groups on the surface.[2]

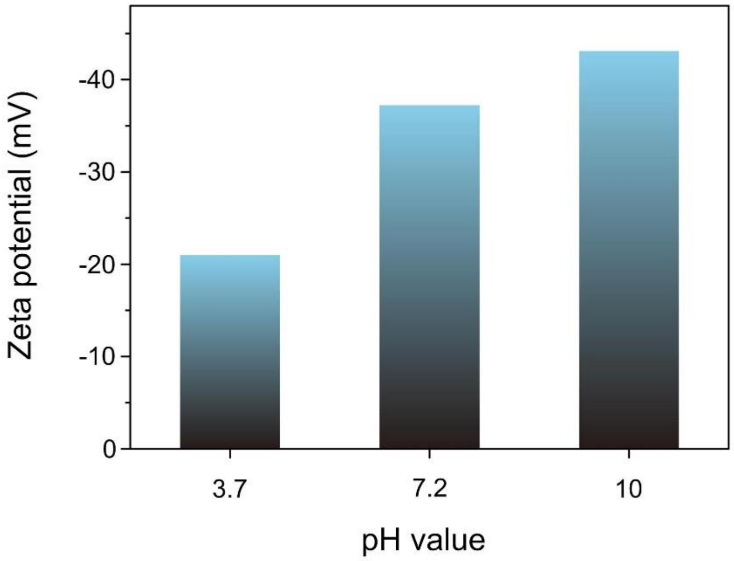

Supplementary Figure 6. Zeta potential values of the ANF membrane under different pH conditions.

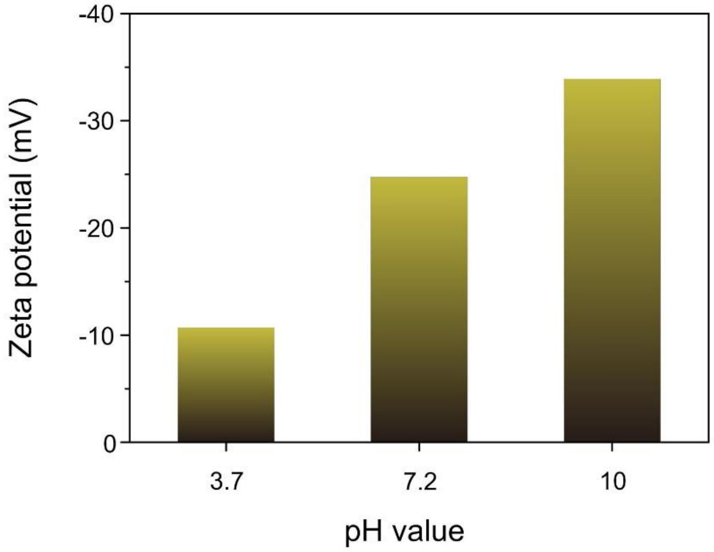

Supplementary Figure 7. Zeta potential values of the polyelectrolyte hydrogel membrane under different pH conditions.

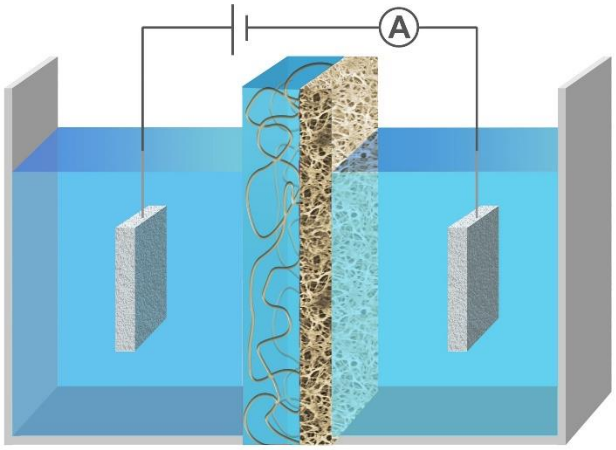

Supplementary Figure 8. Measurement of transmembrane ionic transport. The working electrode is placed in the polyelectrolyte hydrogel membrane side and ground electrode is placed in the ANF membrane side.

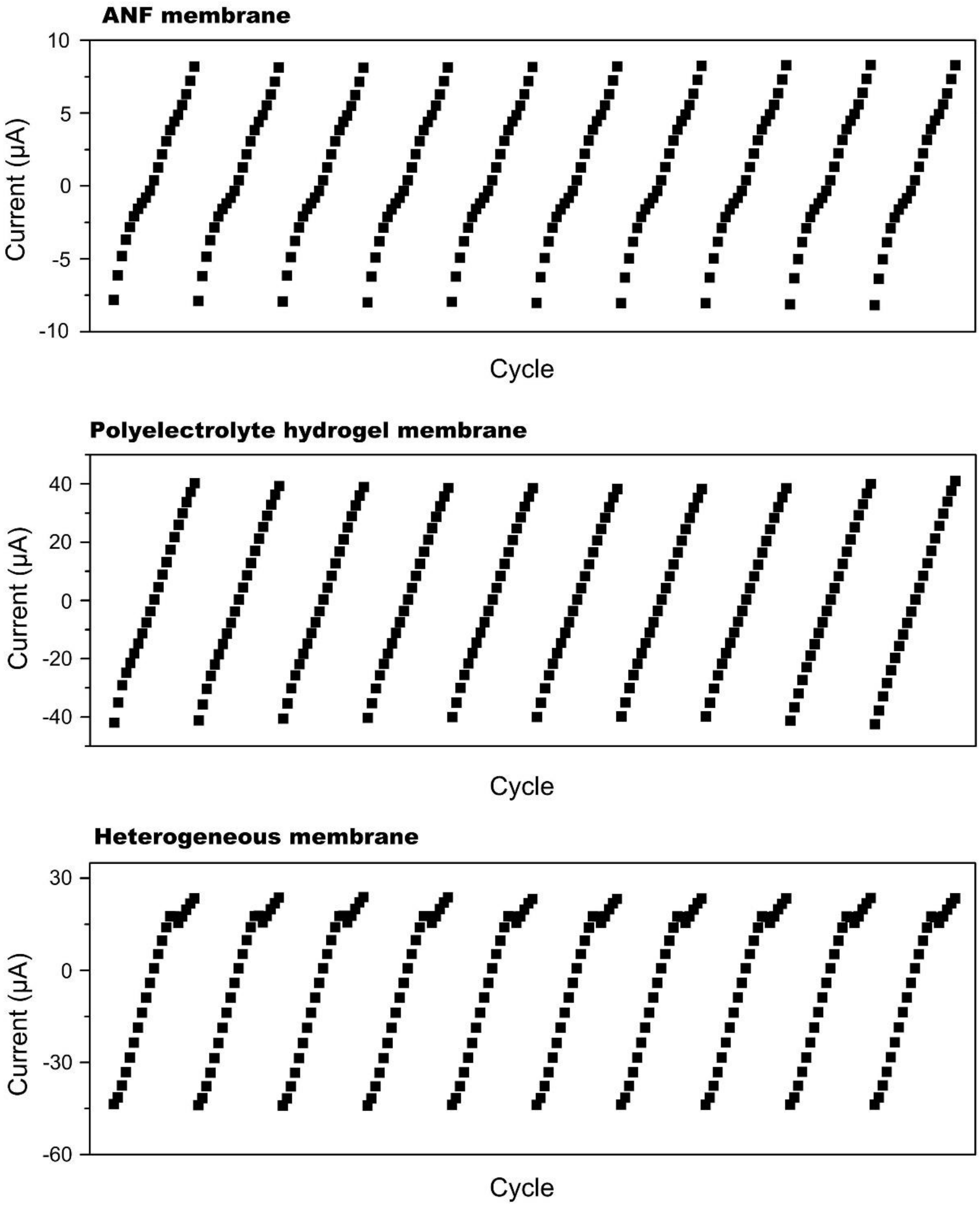

Supplementary Figure 9. The complete *I-V* recordings in the full range of -2 and +2 V in Fig. 3.

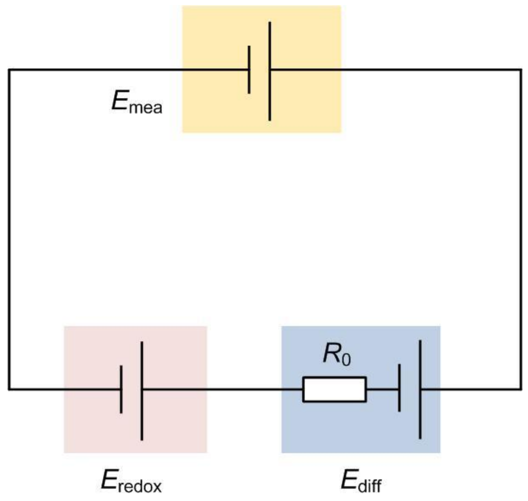

Supplementary Figure 10. Equivalent circuit of the system. The measured open-circuit voltage is composed of the redox potential on the electrode and the diffusion potential contributed by the membranes.[3]

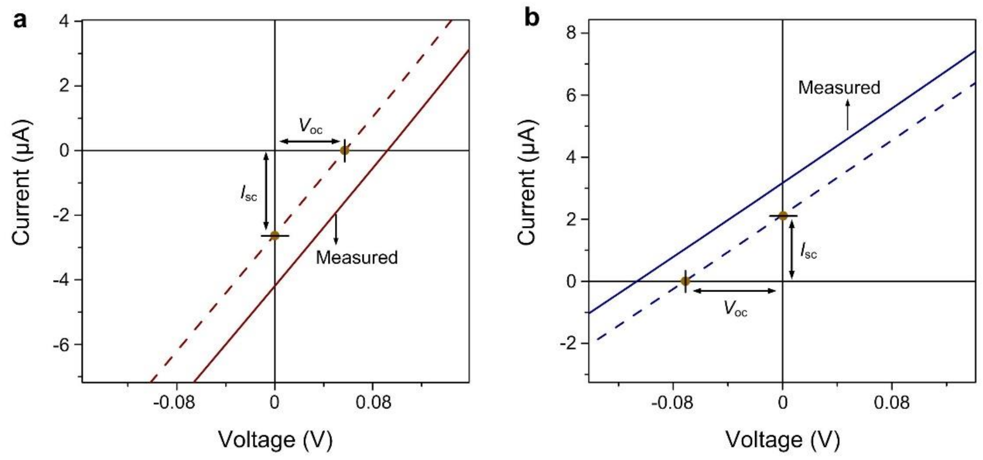

Supplementary Figure 11. *I*-*V* curves under forward (a) and backward (b) concentration gradient before (solid line) and after (dashed line) the subtraction of the E_{redox}. In this work, the value of E_{redox} (~35 mV) was measured experimentally through replacing the hybrid membrane by a non-selective silicon membrane containing a single micro-window in which case the measured potential was contributed solely by E_{redox}. Because the diffusion of ions did not affect the bulk concentration obviously, the electrode potential remained stable during the calibration process. This experimental method could largely preclude the influence brought by many unexpected factors such as the contamination and electrode imperfection. Then the contribution of the E_{diff} can be obtained by subtracting the E_{redox}.[4]

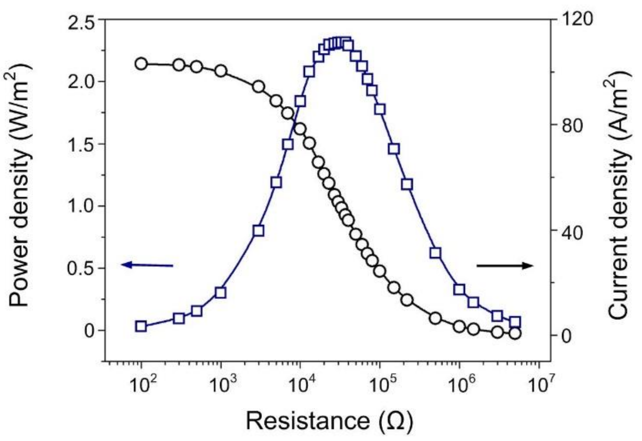

Supplementary Figure 12. Power generation of the heterogeneous membrane under backward concentration gradient.

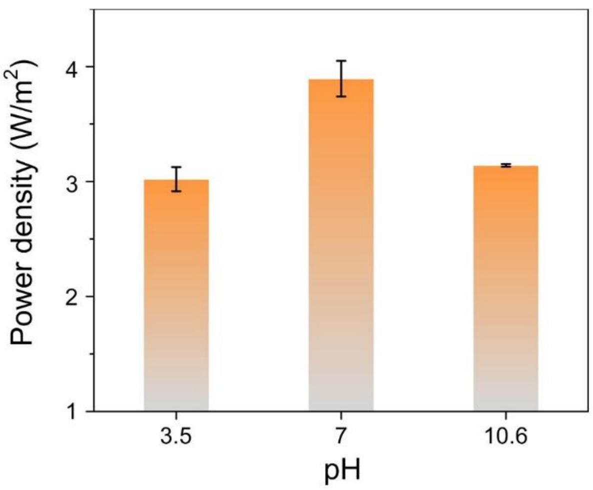

Supplementary Figure 13. Influence of the pH of the electrolyte solution on the power density. Error bars represent s. d. (*n* = 3).

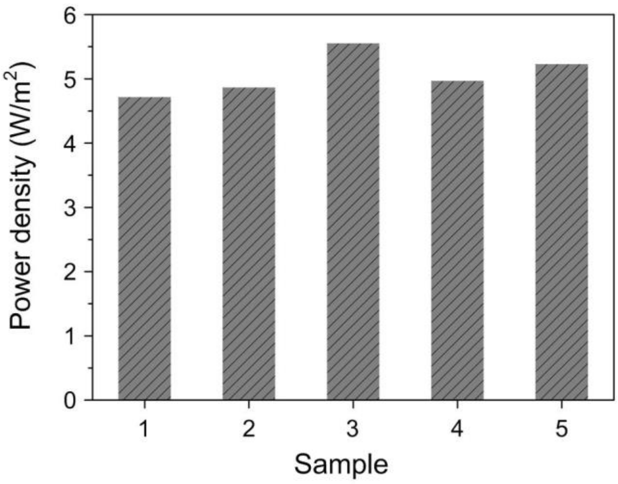

Supplementary Figure 14. The power density of series of optimized membranes when we mix real sea water with river water, achieving a value 5.06 ± 0.33 W/m^2.

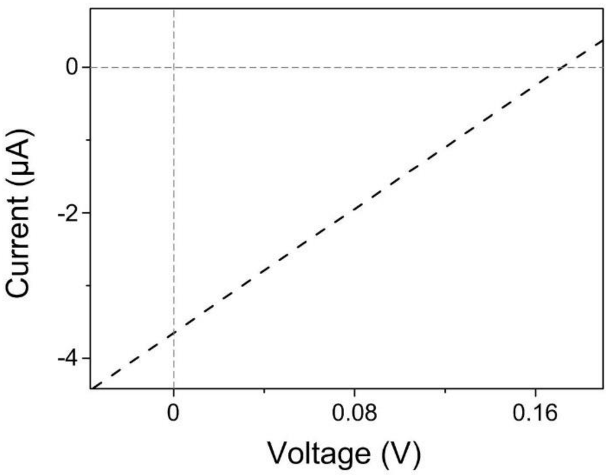

Supplementary Figure 15. *I-V* curve of the heterogeneous membrane under natural river water and sea water.

Supplementary References

[1] Kim, D. K., Duan, C. H., Chen, Y. F. & Majumdar, A. Power generation from concentration gradient by reverse electrodialysis in ion-selective nanochannels. *Microfluid. Nanofluid.* **9**, 1215–1224 (2010).

[2] Patterson, B. A., Sodano, H. A. Enhanced interfacial strength and UV shielding of aramid fiber composites through ZnO nanoparticle sizing. *ACS Appl. Mater. Interfaces* **8**, 33963−33971 (2016).

[3] Feng, J. *et al.* Single-layer MoS_2 nanopores as nanopower generators. *Nature* **536**, 197–200 (2016).

[4] Gao, J. *et al.* High-performance ionic diode membrane for salinity gradient power generation. *J. Am. Chem. Soc.* **136**, 12265–12272 (2014).
